# Complete Genome Sequence of Akkermansia muciniphila JCM 30893, Isolated from Feces of a Healthy Japanese Male

**DOI:** 10.1128/MRA.01543-19

**Published:** 2020-02-13

**Authors:** Yusuke Ogata, Mitsuo Sakamoto, Moriya Ohkuma, Masahira Hattori, Wataru Suda

**Affiliations:** a Laboratory for Microbiome Sciences, RIKEN Center for Integrative Medical Sciences, Yokohama, Japan; b Microbe Division/Japan Collection of Microorganisms, RIKEN BioResource Research Center, Tsukuba, Ibaraki, Japan; c PRIME, Japan Agency for Medical Research and Development (AMED), Tsukuba, Ibaraki, Japan; d Graduate School of Advanced Science and Engineering, Waseda University, Tokyo, Japan; University of Maryland School of Medicine

## Abstract

Akkermansia muciniphila is an anaerobic and mucin-degrading bacterium in the human gut. Here, we report the complete genome sequence of Akkermansia muciniphila JCM 30893 harboring the plasmid pJ30893.

## ANNOUNCEMENT

Akkermansia muciniphila is an anaerobic and Gram-negative bacterium and was first isolated from human feces (1). *A. muciniphila* degrades and utilizes mucin in the human intestine and was also reported to have associations with several diseases, including obesity and diabetes (2–4). Here, we report the genome of a strain, *A. muciniphila* JCM 30893, isolated from feces of a 45-year-old healthy Japanese male.

A total of 0.5 g of a fecal sample was suspended in 4.5 ml of prereduced phosphate-buffered saline (PBS). Each dilution of a fresh fecal sample was plated onto Columbia blood agar supplemented with 5% (vol/vol) horse blood. After 2 to 4 days of incubation at 37°C under a H_2_-CO_2_-N_2_ (1:1:8 [vol/vol/vol]) gas mixture, strain CBH12S (= JCM 30893) was isolated. Genomic DNA extraction, sequencing with the Illumina MiSeq and PacBio Sequel platforms, quality checking of reads, *de novo* hybrid assembly of both reads, and quality checking of the genome were performed as previously described (5), and default parameters were used for all software unless otherwise specified. We obtained a total of 278,916,914 bases from 467,638 filter-passed Illumina paired reads with an average length of 298.2 bp, and a total of 676,968,912 bases from 43,760 filter-passed PacBio reads with an average length of 15,470 bp. The assembly generated two circular single contigs, corresponding to the *A. muciniphila* JCM 30893 chromosome and the plasmid pJ30893. The ratio of the average read depth of the two contigs estimated the copy number of pJ30893 to be ∼3 per chromosome, which was estimated by mapping the reads to contigs using minimap2 (v. 2.13-r850) (6).

The *A. muciniphila* JCM 30893 chromosome was 2,845,645 bp long, with a G+C content of 55.6%, and encoded 2,332 protein-coding genes and 54 tRNA, 3 5S rRNA, 3 16S rRNA, and 3 23S rRNA genes. pJ30893 was 32,814 bp long, with a G+C content of 54.6%, and encoded 43 protein-coding genes. The quality was checked using CheckM (v. 1.0.11) (7), estimating the genome completeness and contamination of the *A. muciniphila* JCM 30893 chromosome to be 98.0% and 0.68% without strain heterogeneity, respectively. The average and the highest nucleotide identity of the *A. muciniphila* JCM 30893 chromosome was 98.9% with the published *A. muciniphila* DSM 22959^T^ chromosome using ANI Calculater (8). In contrast, pJ30893 had no significant similarity with any genome in the publicly available databases. A similarity search of the 43 genes in pJ30893 against the Clusters of Orthologous Groups of proteins (COGs) database (v 2014 update) (9) using blastp (v. 2.6.0+) hit eight COGs with an E value of ≤0.00001, of which four (COG0582, COG1783, COG3600, and COG4388) were assigned as category X (mobilome). A similarity search against the Prokaryotic Virus Orthologous Groups (pVOG) database (v. 2016 update) (10) using blastp also hit nine VOGs with an E value of ≤0.00001, including VOG1298 with 65.1% identity and 95.5% coverage. These data suggested that pJ30893 was a plasmid.

### Data availability.

The complete genome sequence of *A. muciniphila* JCM 30893 was deposited in DDBJ/ENA/GenBank under the accession no. AP021898 and AP021899, which are linked to the BioProject accession no. PRJDB8988, the BioSample accession no. SAMD00192834, and the DDBJ Sequence Read Archive (SRA) accession no. DRX188527 and DRX188528.
